# Unilateral Vision Loss in a Child Revealing Ocular Toxocariasis

**DOI:** 10.7759/cureus.99150

**Published:** 2025-12-13

**Authors:** Oumaima El Korno, Zineb Hilali, Samira Tachfouti, Abdellah Amazouzi, Lalla Ouafa Cherkaoui

**Affiliations:** 1 Ophtalmology, Ibn Sina Hospital, Rabat, MAR; 2 Faculty of Medicine, Hopital des Specialités de Rabat, Rabat, MAR; 3 Ophtalmology A, Hopital des Specialités de Rabat, Rabat, MAR; 4 Ophthalmology A, Hopital des Specialités de Rabat, Rabat, MAR; 5 Faculty of Medicine, Centre Hospitalier Universitaire (CHU) Ibn Sina, Rabat, MAR; 6 Ophthalmology A, Hospital of Specialties, Mohammed V University, Rabat, MAR

**Keywords:** ocular toxocariasis, pars plana vitrectomy, pediatric uveitis, peripheral granuloma, retinal vasculitis, vitreoretinal traction

## Abstract

Ocular toxocariasis is a parasitic infection caused by *Toxocara canis* or *T. cati*, typically affecting children and presenting as unilateral vision loss. This report describes a rare presentation of peripheral retinal vasculitis, a peripheral granuloma, and vitreoretinal traction in a seven-year-old child.

A seven-year-old male presented with progressive visual loss in his left eye over three months. Ophthalmological examination revealed a best-corrected visual acuity of 20/20 in the right eye and counting fingers in the left eye. Fundus examination of the left eye showed peripheral retinal vasculitis, a yellow-white peripheral granuloma, and a fibrous vitreoretinal band extending from the lesion toward the posterior pole. B-scan ultrasonography confirmed vitreous condensation and focal retinal elevation. Serological testing for Toxocara (ELISA) was positive. The patient was treated with oral albendazole and systemic corticosteroids, followed by pars plana vitrectomy due to persistent vitreoretinal traction. At one-month follow-up, retinal architecture had stabilised, but visual acuity in the affected eye showed no improvement. This case highlights an uncommon presentation of ocular toxocariasis with peripheral retinal vasculitis and vitreoretinal traction, stressing the importance of early diagnosis and combined medical-surgical management to preserve visual function.

## Introduction

Ocular toxocariasis (OT) is a zoonotic parasitic disease caused by the larvae of *Toxocara canis* (dog roundworm) or *T. cati* (cat roundworm), which are common intestinal parasites in canids and felids. Transmission occurs via ingestion of embryonated eggs from contaminated soil, sandboxes, or undercooked meat, primarily affecting children aged 2-10 years due to geophagia or poor hygiene. Larvae hatch in the intestine, penetrate the gut wall, and migrate hematogenously to various organs, including the eye, where they trigger a vigorous intraocular inflammatory response. OT typically presents unilaterally with diverse manifestations, including posterior pole granuloma, peripheral granuloma, chronic endophthalmitis, or tractional retinal detachment. Peripheral retinal vasculitis remains rare and underreported. This case report aims to detail the diagnostic and management challenges of a rare triad of peripheral granuloma, retinal vasculitis, and significant vitreoretinal traction in unilateral OT, a constellation infrequently described in the literature [1].

## Case presentation

A seven-year-old previously healthy boy presented to our ophthalmology department with a three-month history of progressive visual loss in his left eye. There was no reported ocular trauma or systemic illness, but he had regular contact with household dogs. On examination, visual acuity was 20/20 in the right eye and limited to counting fingers in the left eye. The left pupil exhibited a relative afferent pupillary defect. Anterior segment examination was unremarkable in both eyes.

**Figure 1:**
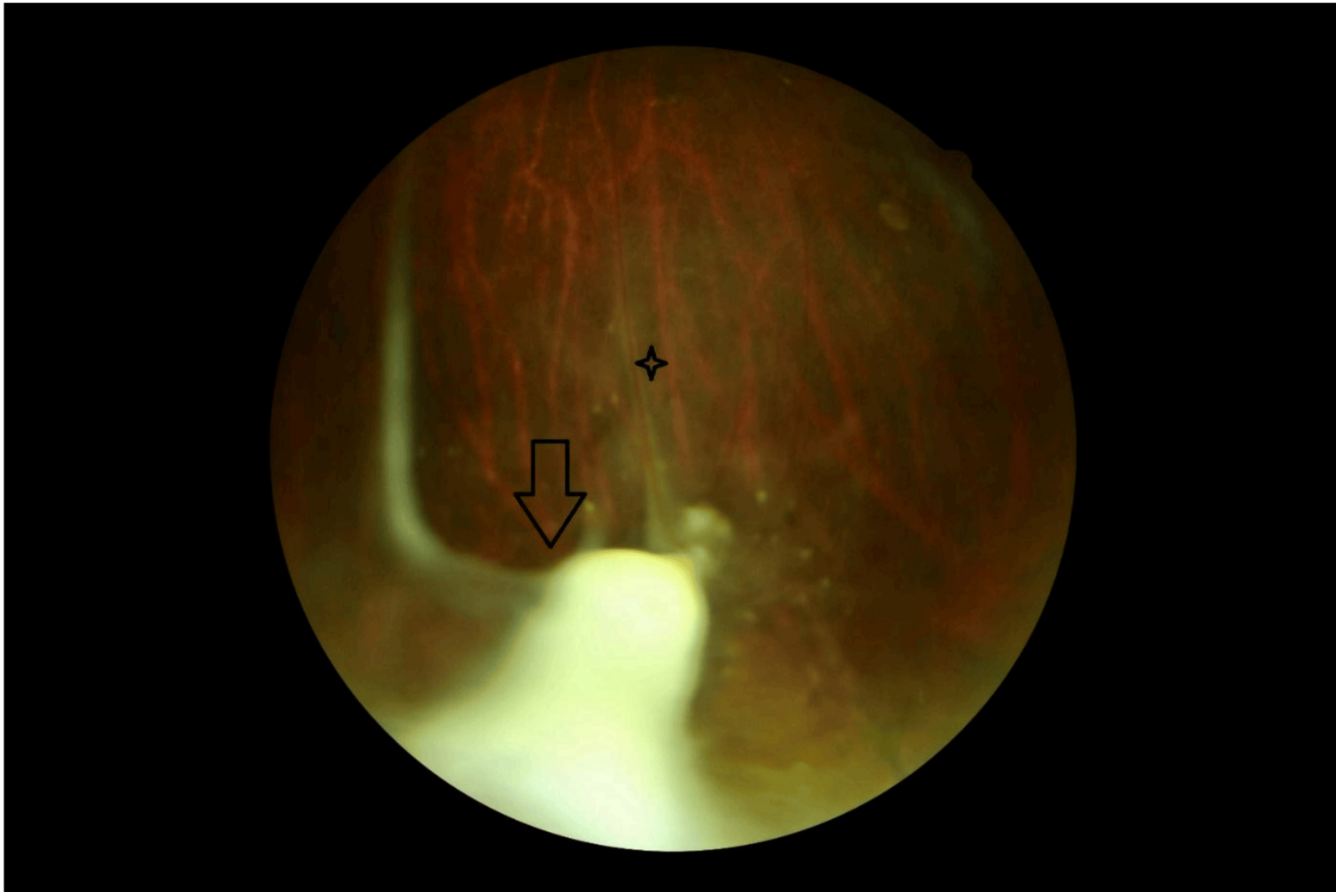
Fundus examination revealing a peripheral inferotemporal granuloma (arrow) associated with a fibrous band and vascular sheathing (star).

**Figure 2:**
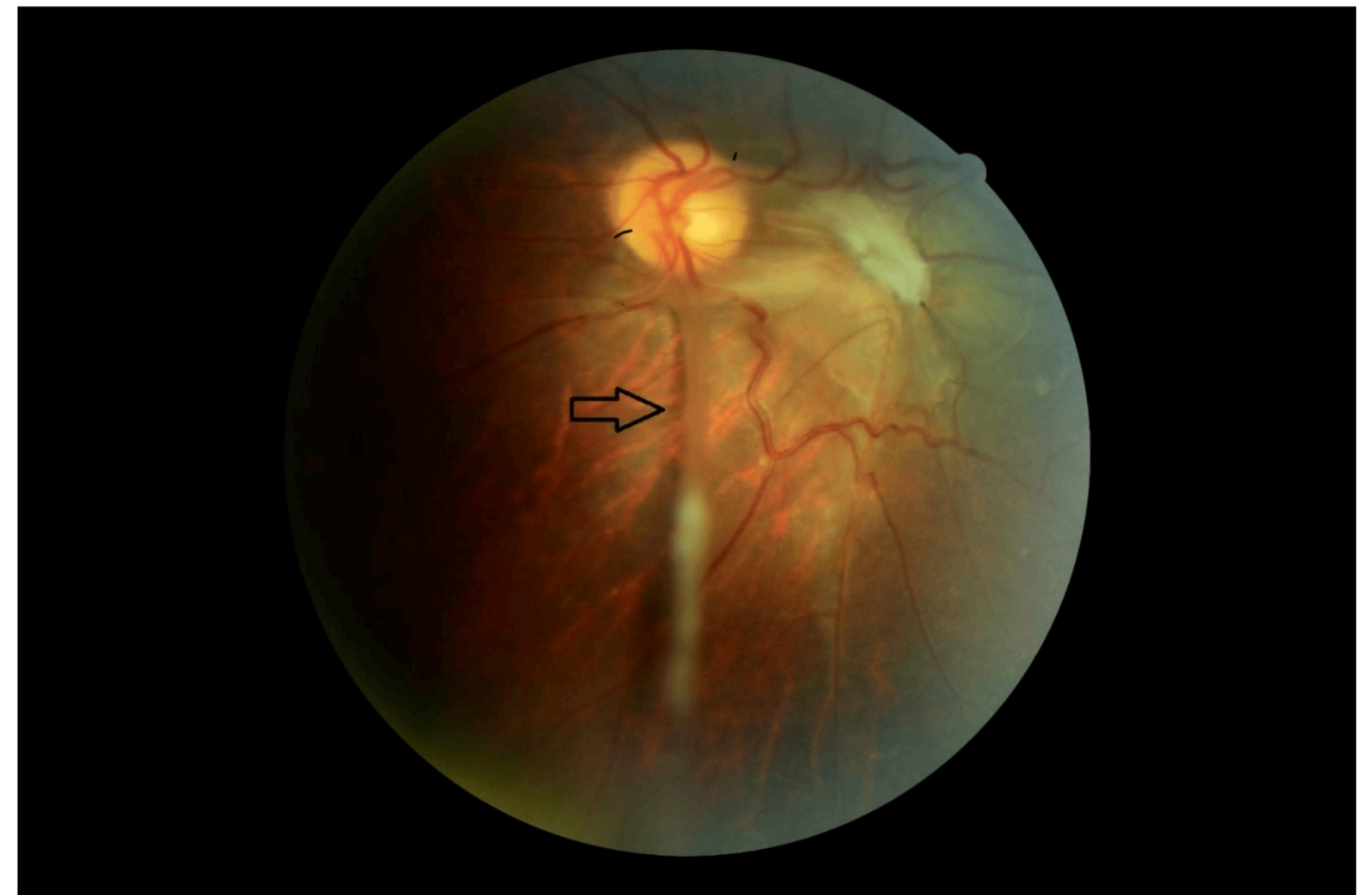
A dense fibrous vitreoretinal band extending from the inferotemporal lesion to the optic disc, resulting in evident retinal traction (arrow).

**Figure 3:**
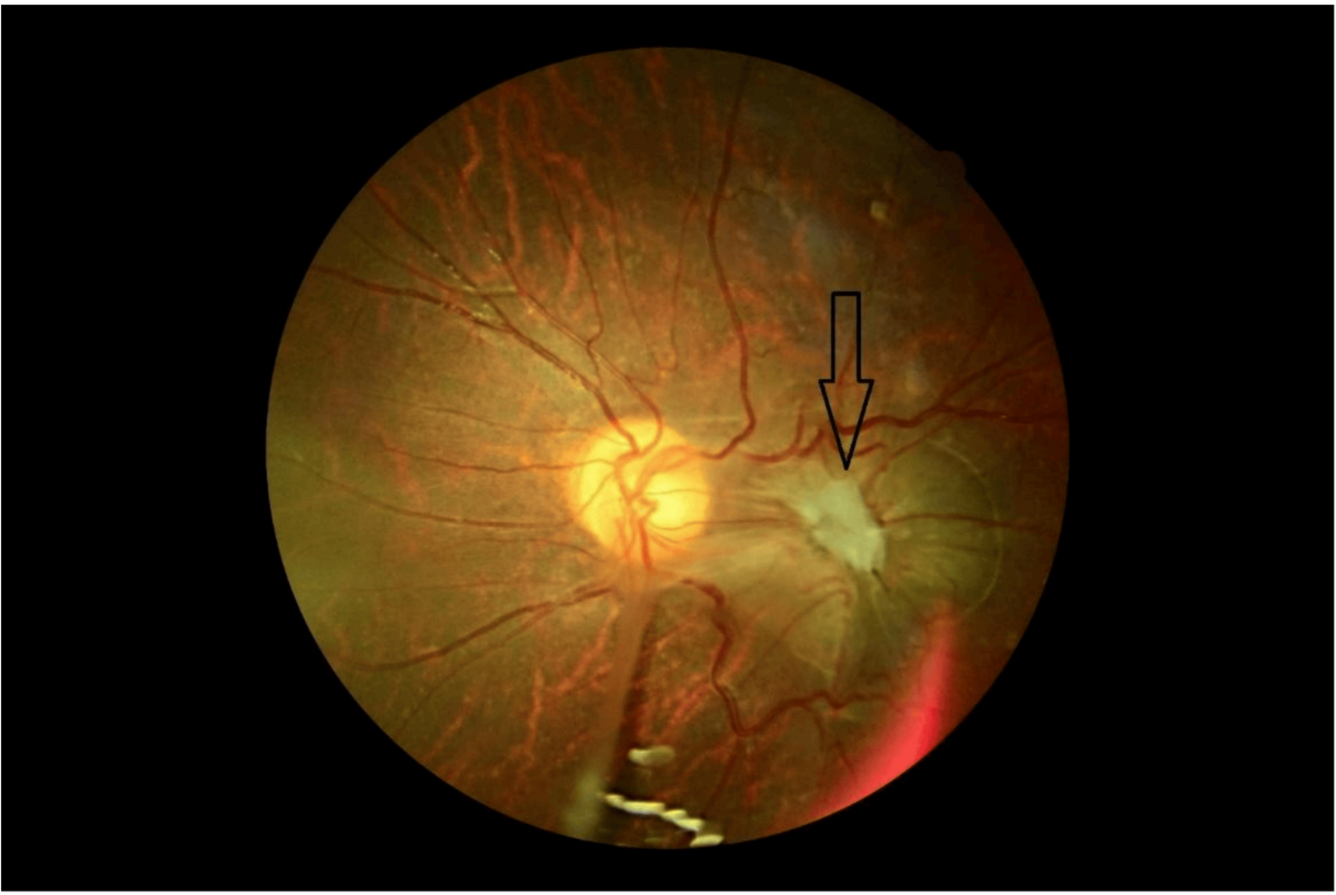
Fundus image showing submacular fibrosis (arrow) in ocular toxocariasis.

Retinography demonstrated a yellowish-white granulomatous lesion in the inferotemporal peripheral retina (Figure 1). A dense vitreoretinal fibrous band extended from the lesion toward the optic disc, causing visible retinal traction (Figure 2). Additionally, submacular fibrosis was noted at the macula, with evidence of dragged and tortuous retinal vessels (Figure 3).

**Figure 4:**
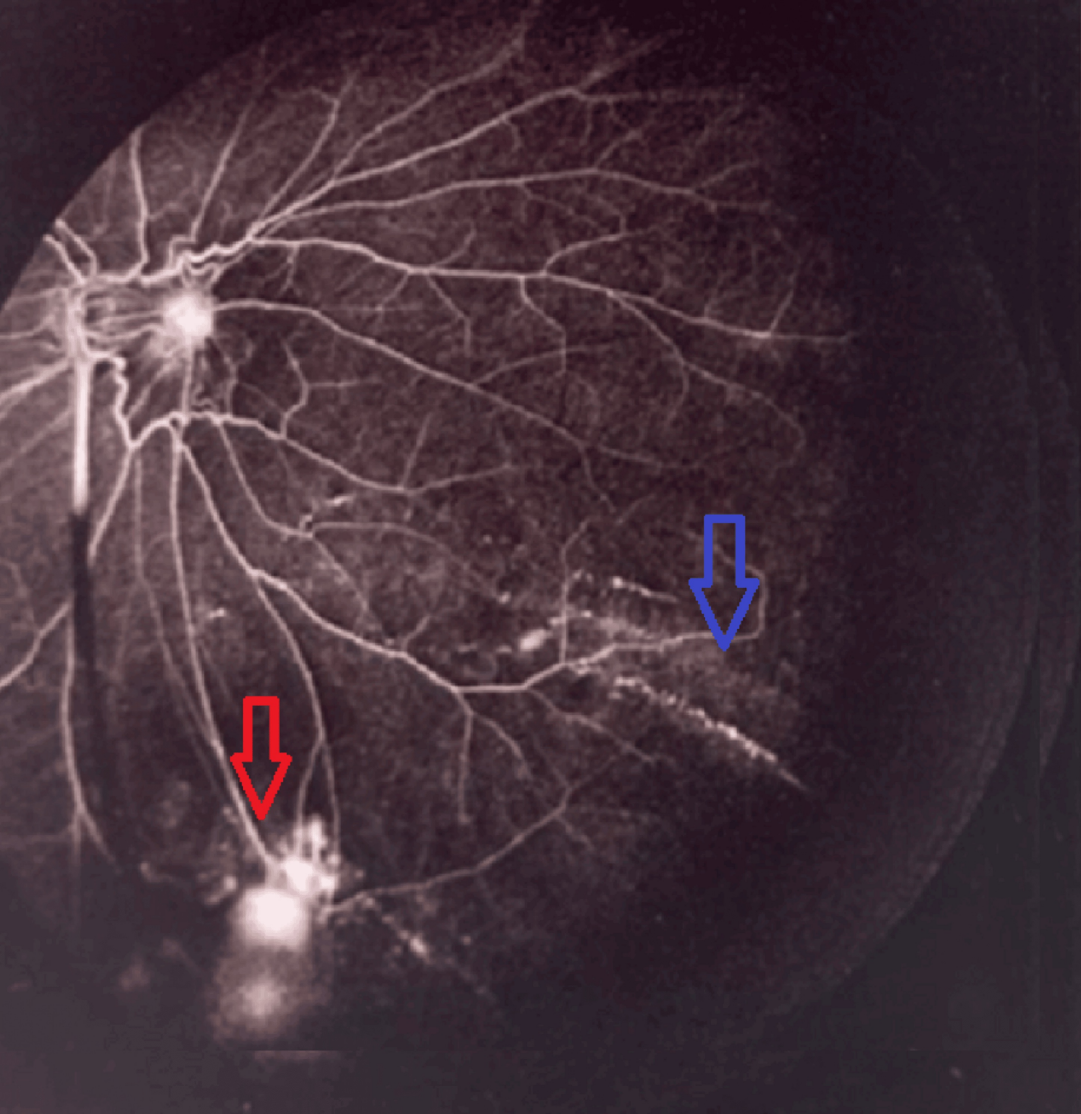
Active peripheral retinal vasculitis (blue arrow) with dye leakage and staining, predominantly in the inferior quadrant, corresponding to retinal neovascularization (red arrow).

**Figure 5:**
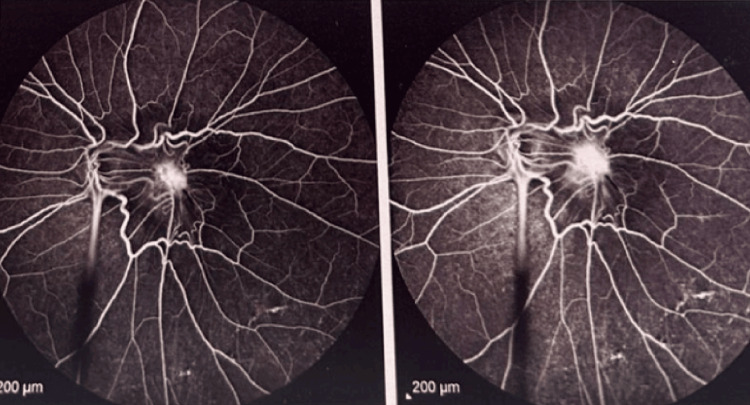
Diffuse macular leakage indicative of macular edema and inflammatory activity.

Fluorescein angiography revealed active peripheral retinal vasculitis with dye leakage and staining, particularly in the inferior quadrant, corresponding to an area of retinal neovascularization (Figure 4). Diffuse macular leakage was also present, consistent with associated macular edema and inflammation (Figure 5).

**Figure 6:**
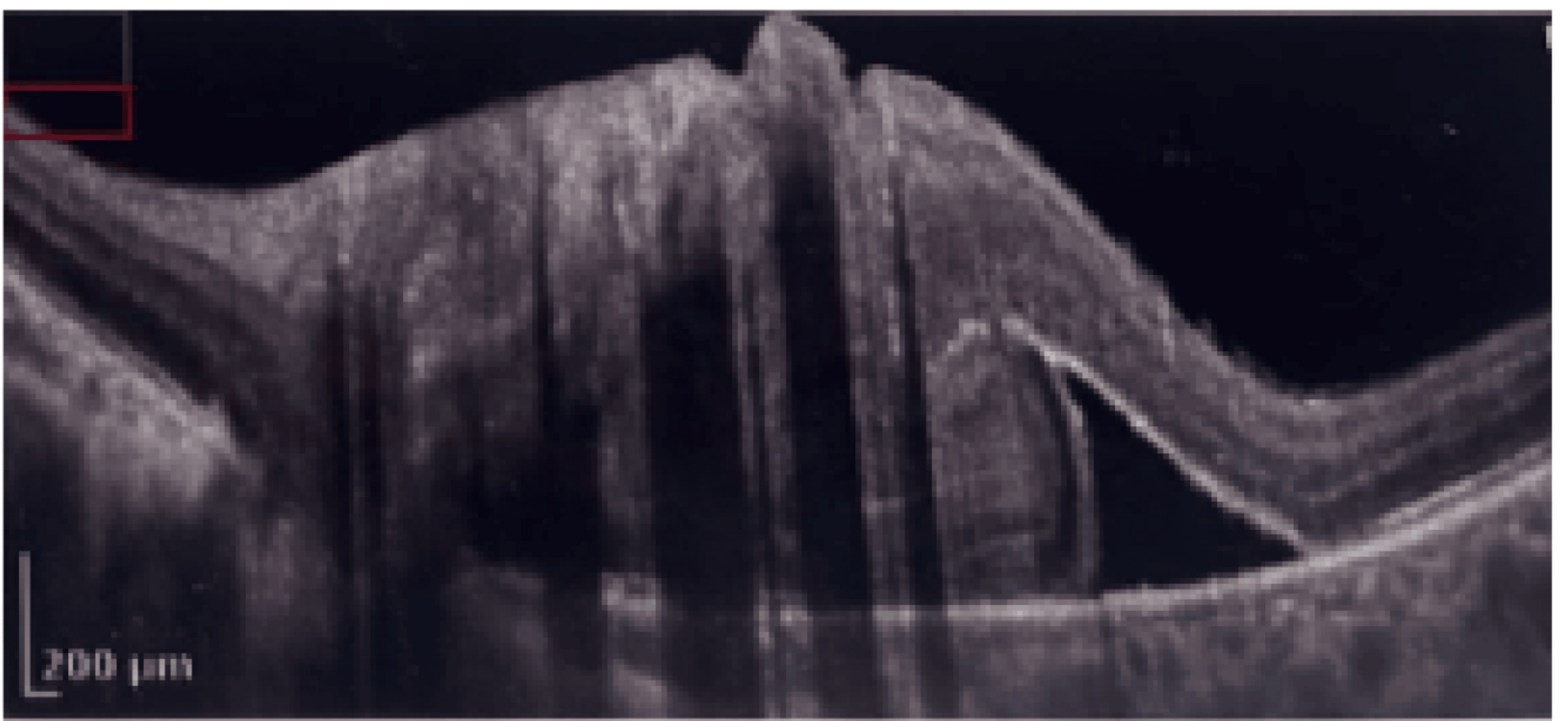
Serous retinal detachment with associated fibrotic epiretinal membrane on macular OCT.

Optical coherence tomography (OCT) of the macula revealed a serous retinal detachment associated with a fibrotic epiretinal membrane, causing traction and macular thickening (Figure 6).

**Figure 7:**
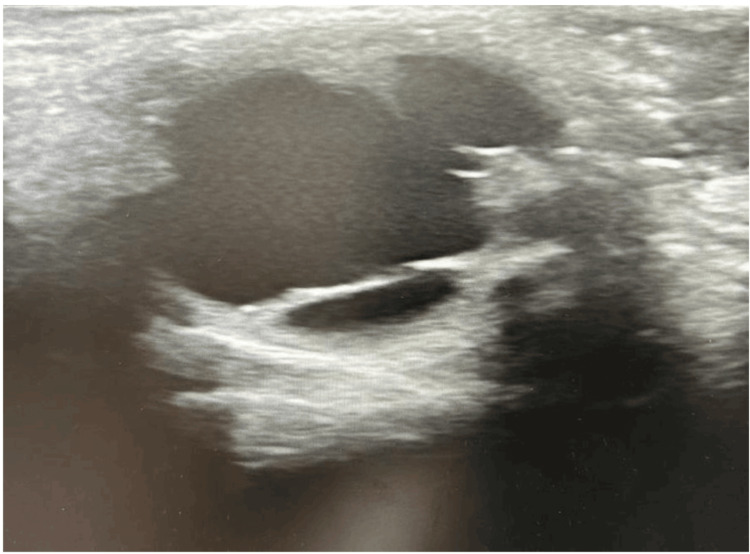
Ocular ultrasound showing localized retinal elevation consistent with vitreoretinal traction.

The right eye was entirely normal. B-scan ultrasonography of the left eye demonstrated focal vitreous opacities and a localized area of retinal elevation, consistent with vitreoretinal traction (Figure 7).

Blood work showed mild peripheral eosinophilia, and enzyme-linked immunosorbent assay (ELISA) for Toxocara canis IgG was positive. The child was treated with oral albendazole at a dose of 15 mg/kg/day for 10 days, combined with a tapering course of oral prednisone (1 mg/kg/day). Despite medical therapy, the fibrocellular band persisted with evidence of worsening tractional distortion of the posterior pole. Pars plana vitrectomy was subsequently performed to relieve traction and prevent further macular damage. At one-month follow-up, retinal architecture had stabilized, but visual acuity in the affected eye showed no improvement. No signs of recurrent inflammation or new lesions were observed at six months.

## Discussion

Ocular toxocariasis is a major cause of unilateral visual impairment in children, particularly in regions with high rates of soil contamination and close contact with stray or inadequately dewormed dogs and cats. It results from hematogenous migration of Toxocara larvae to the eye after ingestion of embryonated eggs, where they induce a granulomatous inflammatory response with potential for severe structural damage [1].

In most pediatric cases, ocular toxocariasis presents as unilateral decreased visual acuity with posterior pole granuloma, peripheral granuloma, chronic endophthalmitis, or tractional retinal detachment. Peripheral retinal vasculitis, however, is rarely emphasized in the literature and may mimic other infectious or inflammatory entities, such as toxoplasmosis, sarcoidosis, or idiopathic retinal vasculitis. In the present case, the association of peripheral granuloma, peripheral retinal vasculitis, and marked vitreoretinal traction represents an uncommon constellation that can complicate both diagnosis and management [1].

The presentation of ocular toxocariasis can vary significantly depending on the location and extent of the larval infection. In children, the disease typically manifests as unilateral vision loss, although bilateral involvement has been reported. The most common symptom is a decrease in visual acuity, often accompanied by signs of inflammation, such as retinal granulomas, vitreous opacities, or retinal detachment. The condition can present with leukocoria or strabismus, particularly in younger children. However, these findings are not exclusive to toxocariasis and can overlap with other serious ocular conditions, such as retinoblastoma or endophthalmitis, which makes accurate diagnosis challenging [1].

The diagnostic process in this child was driven by the combination of clinical context and ancillary testing. Unilateral visual loss, peripheral yellow-white granuloma, vasculitis, and vitreoretinal traction raised suspicion for an infectious or inflammatory etiology. B-scan ultrasonography showing vitreous condensation and focal retinal elevation, together with positive Toxocara serology (ELISA), supported the diagnosis of ocular toxocariasis in the absence of signs suggesting alternative diagnoses, such as retinoblastoma or endophthalmitis. This case underlines the importance of systematically considering ocular toxocariasis in children with unexplained unilateral uveitis, granuloma, or vasculitis, especially in the presence of environmental exposure to dogs or contaminated soil [2].

Ocular toxocariasis remains a diagnostic challenge due to its varied presentations. In this case, peripheral retinal vasculitis was associated with granulomatous inflammation and vitreoretinal traction, mimicking other infectious or inflammatory conditions such as toxoplasmosis or sarcoidosis. ELISA serology and characteristic fundus findings supported the diagnosis [2].

When evaluating a case of ocular toxocariasis in a pediatric patient, several other conditions must be considered to differentiate it accurately from similar ocular pathologies. These include retinoblastoma, endophthalmitis, chorioretinitis, ocular histoplasmosis, and other parasitic infections [3,4].

Management of ocular toxocariasis generally combines antiparasitic therapy to target viable larvae and systemic corticosteroids to control inflammation. In this patient, oral albendazole was administered at a dose of 15 mg/kg/day for ten days, together with systemic corticosteroids (oral prednisone ten mg/kg/day). This regimen aimed to reduce parasite burden and modulate the inflammatory response before surgical intervention. Despite medical therapy, significant vitreoretinal traction persisted, justifying pars plana vitrectomy to relieve traction, clear vitreous opacities, and stabilize the retinal architecture. The discussion of anti-VEGF (vascular endothelial growth factor) therapy, although of academic interest in inflammatory macular edema or neovascularization, is not directly relevant to the management undertaken in this case and has therefore been omitted to maintain focus on the actual therapeutic choices [5,6].

While the use of anti-VEGF therapy in ocular toxocariasis is still under exploration, it appears to be a promising adjunct treatment, particularly for managing macular edema, vascular leakage, and neovascularization resulting from the chronic inflammatory response associated with the infection. Anti-VEGF agents, such as bevacizumab, ranibizumab, and aflibercept, have shown potential in reducing retinal fluid and improving vision, but they do not address the underlying parasitic infection [7]. Antiparasitic therapy, such as albendazole, remains essential for the treatment of the infection itself [6,7].

Posterior vitrectomy has been employed in managing ocular toxocariasis, particularly for complications such as retinal detachment, epiretinal membranes, and vitreous opacities [8].

The postoperative course in this child illustrates a key prognostic point. Although vitrectomy and combined medical treatment allowed anatomical stabilization with relief of traction, visual acuity did not improve. The most plausible explanation is longstanding macular involvement with established submacular fibrosis and chronic tractional changes at the time of presentation. This aligns with previous reports indicating that delayed diagnosis and advanced structural damage substantially limit visual recovery, even when anatomical outcomes are favorable.

Overall, this case highlights several practical messages for clinicians: ocular toxocariasis should be actively considered in children with unilateral peripheral granuloma and vasculitis; detailed documentation of antiparasitic and corticosteroid regimens is essential to guide future management; and in delayed presentations with advanced macular damage, realistic goals may shift from visual recovery to anatomical stabilization and prevention of further complications [8].

## Conclusions

Ocular toxocariasis should be considered in children with unilateral vision loss and peripheral granulomas. Peripheral vasculitis and vitreoretinal traction, though rare, can lead to significant visual impairment. In delayed pediatric ocular toxocariasis with advanced vitreoretinal traction and macular involvement, combined antiparasitic, steroid, and vitrectomy management achieves anatomical stabilization but rarely restores vision. Early diagnosis before structural damage remains critical. Given the potential for recurrence, ongoing monitoring and education regarding preventive measures, such as reducing exposure to soil and stray animals, are essential in reducing the incidence of this parasitic infection in children.
